# Adversities and mental health needs of pregnant adolescents in Kenya: identifying interpersonal, practical, and cultural barriers to care

**DOI:** 10.1186/s12905-018-0581-5

**Published:** 2018-06-15

**Authors:** Judith Osok, Pius Kigamwa, Keng-Yen Huang, Nancy Grote, Manasi Kumar

**Affiliations:** 1 Department of Psychiatry, School of Medicine College of Health Sciences, University of Nairobi, P. O. Box 20386-00100, Nairobi, Kenya; 2 Department of Psychiatry, School of Medicine College of Health Sciences, University of Nairobi, P. O. Box19676-00202, Nairobi, Kenya; 3 Department of Public Health and Child and Adolescent Psychiatry, New York University, New York, NY 10016 USA; 4 Department of Social Work, University of Washington, 4101 15th Avenue NE, Seattle, WA 98105-6250 USA; 5 Department of Psychiatry, College of Health Sciences, University of Nairobi, P.O Box 47074-00100, Nairobi, Kenya

**Keywords:** Pregnant adolescents, Depression, Parenting, Poverty, Stigma, Kenya

## Abstract

**Background:**

Adolescent pregnancies present a great public health burden in Kenya and Sub-Saharan Africa (UNFPA, Motherhood in Childhood: Facing the challenge of Adolescent Pregnancy, 2013). The disenfranchisement from public institutions and services is further compounded by cultural stigma and gender inequality creating emotional, psychosocial, health, and educational problems in the lives of vulnerable pregnant adolescents (Int J Adolesc Med Health 15(4):321–9, 2003; BMC Public Health 8:83, 2008). In this paper we have applied an engagement interview framework to examine interpersonal, practical, and cultural challenges faced by pregnant adolescents.

**Methods:**

Using a qualitative study design, 12 pregnant adolescents (ages 15–19) visiting a health facility’s antenatal services in Nairobi were interviewed. All recruited adolescents were pregnant for the first time and screened positive on the nine-item Patient Health Questionnaire (PHQ-9) with 16% of 176 participants interviewed in a descriptive survey in the same Kangemi primary health facility found to be severely depressed (Osok et al., Depression and its psychosocial risk factors in pregnant Kenyan adolescents: a cross-sectional study in a community health Centre of Nairobi, BMC Psychiatry, 2018 18:136 https://doi.org/10.1186/s12888-018-1706-y). An engagement interview approach (Social Work 52(4):295–308, 2007) was applied to elicit various practical, psychological, interpersonal, and cultural barriers to life adjustment, service access, obtaining resources, and psychosocial support related to pregnancy. Grounded theory method was applied for qualitative data sifting and analysis (Strauss and Corbin, Basics of qualitative research, 1990).

**Results:**

Findings revealed that pregnant adolescents face four major areas of challenges, including *depression, anxiety and stress around the pregnancy, denial of the pregnancy*, *lack of basic needs provisions and care, and restricted educational or livelihood opportunities for personal development post pregnancy*. These challenges were related both to existing social and cultural values/norms on gender and traditional family structure, as well as to service structural barriers (including prenatal care, mental health care, newborn care, parenting support services). More importantly, dealing with these challenges has led to negative mental health consequences in adolescent pregnant girls, including feeling insecure about the future, feeling very defeated and sad to be pregnant, and feeling unsupported and disempowered in providing care for the baby.

**Conclusions:**

Findings have implications for service planning, including developing more integrated mental health services for pregnant adolescents. Additionally, we felt a need for developing reproductive education and information dissemination strategies to improve community members’ knowledge of pregnant adolescent mental health issues.

**Electronic supplementary material:**

The online version of this article (10.1186/s12905-018-0581-5) contains supplementary material, which is available to authorized users.

## Background

Adolescent pregnancy is not only a reproductive health issue but also a cause and an outcome of shifting socio-cultural practices around family rearing, social exclusion of the girl child. This disenfranchisement is compounded by weak institutional, legal, and social protection [1–3]. Adolescent age bracket, as defined by the United Nations, is the ages between 10 and 19 years. The adolescent population worldwide is 1.2 billion as estimated by UNICEF [4]. Of these 1.2 billion or so 10–19 year old worldwide, 70% live in developing countries and about 23% in Sub-Saharan Africa (SSA) with the rate of sexual initiation continuing to rise in most of these countries [4]. The UNICEF report further states that SSA is the only region in the world where the adolescent population continues to expand and is projected to have the greatest number of adolescents of any region by 2050. Yet this is also the most challenging place for an adolescent to live in terms of high rates of poverty, gender-based violence, disability, stigma, and discriminatory laws that may curtail adolescents’ access to services, including HIV prevention and treatment, education, assistance in humanitarian emergencies, maternal health, and reproductive care for adolescent girls [4, 5]. There are certain socio-cultural practices that predispose adolescents to risky sexual behaviors: adolescent sleeping arrangements, funeral ceremonies, replacing a deceased married daughter with her younger sister in marriage, widow inheritance among boys, early marriage among girls, and preference for boys/sons [6]. A number of studies have highlighted that poverty exacerbates these practices. With changing nature of family ties due to urbanization, the social protection that these mechanisms offered previously no longer exists leaving young girls highly vulnerable and exposed to structural violence starting from the family itself.

### Reproductive health and adolescent pregnancy in LMICs

Early sexual activity among adolescents in developing countries has been a critical global health problem [4, 7]. It not only contributes to high prevalence of adolescent pregnancy, but also poses challenges in HIV/AIDS prevention, high secondary school dropout in females, and poor adolescent population well-being [8]. A School Health Survey data from WHO showed that *37% of 11–16 years old Kenyan children had ever had sex, 56% of children had sex by 16 years old, and only 45% of children had ever used condom during intercourse* [8]. In Kenya, similar to other SSA countries, 22% of population were adolescents, 56% of adolescents had sex by age 16, only 45% of adolescents used condom during intercourse, 26% of girls give birth by age 18 (or 30% in urban regions), and only 42–51% of adolescents had comprehensive knowledge of HIV [9]. Most global effort in adolescent reproductive health has been focused on HIV/AIDS prevention, with very little attention on marginalized adolescent girls, especially pregnant girls. To effectively address adolescent pregnancy issue in LMICs and develop public health strategies to reduce burden of adolescent health, a better understanding of adolescent pregnancy context, challenges faced by pregnant adolescents, and impact on their well-being is sorely needed. *This study addresses these knowledge and experiential gaps by focusing on adolescent pregnant girls in Kenya and studies challenges faced by this marginalized population.* Findings from qualitative studies like this can become the first step towards developing educational strategies to help adolescents understand the consequences of an unplanned pregnancy and to plan for new services to support pregnant adolescents so as to reduce the negative consequences on this population’s health. The goal of our inquiry is to understand depression and mental health care barriers associated with adolescent pregnancy underscoring the negative social determinants of health that we touched upon during interviews with our participants.

#### Social determinants associated with pregnancies in Kenyan adolescents

The Kenya Ministry of Health found that 18% of young women aged between 15 and 19 have already begun childbearing: 15% are mothers and an additional 3% are pregnant with their first child [10]. Young motherhood is slightly more common in urban areas than in rural areas [11, 12].

- Are pregnant adolescents living in poverty in Kenya more likely than others to be depressed:
  Some of the greatest inequality occurs in the household context where there exists sexual exploitation or physical abuse from within the adolescent’s own families, partners their age or older men. Adolescent depression and mental health burden increases manifold [13]. In a World Bank survey on young people’s health and financial status in Kenya, it was found that most young people are worried about contracting AIDS, getting early pregnancies, getting physically or sexually exploited. These concerns were embedded in larger issues of school completion, worries of whether the current educational and social systems empowered them for employment and stable future ahead [14].
  Depression in perinatal spectrum has recently drawn a lot of attention with a number of studies being conducted in LMIC [15–17] and the psychosocial and health burden of adolescent pregnancies is underscored in each of these reports [18, 19]. Reviewing recent research on mental health outcomes of adolescents in SSA [1, 20–23], we can see that *adolescents in Kenya with little knowledge of contraception, encased by multiple health risks, and limited social protection commonly get pregnant and struggle through their lives; remaining a highly vulnerable underserved population*. *The girls most likely to have a live birth before age 18 reside in rural and remote areas or in urban informal settlements, have little or no education, and live in the poorest households* [23]*.*
  Depression in sub-Saharan Africa is one of the leading contributors to years lived with disability (YLD) and depressive disorders account for 2.87% of the total Disability adjusted life years (DALYs) in Kenyan women of ages 15–49. Unsafe sex and intimate partner violence are amongst the commonly identified risk factors in the Global Burden of Disease estimates associated with DALYS in Kenyan women [24, 25]. UNFPA projects that the potential number for adolescent pregnancies in SSA could eventually equal or surpass the number for South Asia around 2025 to 2030 with a total of 3 million adolescents who could have their first birth much before age 15 in SSA region [26]. Additionally, adolescents lack comprehensive knowledge about the risks stemming from early sexual activity, as well as appropriate measures to prevent infection and pregnancy. Limited access of adolescent mothers to reproductive health services also predisposes them to higher risks of illness and death [6, 27]. Understanding mental health and reproductive health needs and rights of adolescents is therefore central to improving the health services offered to this vulnerable group [28, 29].
- Medical, legislative, and social protection mechanisms:
  KDHS report shows that an estimated half of all pregnancies in women aged 15–19 get terminated or adolescents end up with serious medical complications [9]. Kenya’s Constitution including the 2007 Kenyan reproductive health policy also protects the rights of adolescents to health care and guarantees protection from abuse and neglect, all forms of violence, harmful cultural practices, inhuman treatment and punishment, and hazardous or exploitative labor—Article 53 and access to free youth friendly health services [30]. Another critical gap in this regard is the transition from child health services to adult services as adolescents get caught between this with limited to no adolescent friendly services on ground [31]. *Despite these policy stipulations, on ground, there is limited evidence of this as only 12% of health facilities actually provide the recommended comprehensive RH services to adolescents.*
- Paucity of MH resources and care at primary health facilities: Mental health coverage is more limited with very few practitioners on ground and the paucity of formally employed social workers, community health extension workers, and unskilled and unharnessed potential of the community health volunteers and community health committees makes the adolescent mental health burden even deeper. The significance of adolescent mental health disorders over the lifespan has only recently caught attention and remains a relatively understudied area in global health [32]. Primary health care facilities have taken on the burden of ensuring the adolescent well-being among the local communities. Early intervention and adolescent friendly services have caught attention in high income countries but remain an area requiring considerably more impetus in programming and staff training to deliver care in the LMIC. Evaluation studies done worldwide have noted that very limited interventions have been tested to address adolescent mental health [33] and the few that have been used, their effectiveness for high risk or for differential cultural/socioeconomic contexts have not been demonstrated.
- Sociocultural practices and their impact on communities and schools: The context of early pregnancy presents the adolescent with damaging sociocultural repercussions [34]. Kenya is a religious society with a strong presence of Catholic Church, various protestant denominations, including very active evangelical groups, and Muslim organizations are most organized and politically influential civil-society stakeholders. Although increasingly these religious groups are being used to spread sexual and reproductive health information, historically these groups favored prevention messages propagating abstinence until marriage [6]. Primary school children in Kenya are ‘not supposed’ to be sexually active thus the school health policies do not actively promote sex education. Girls who become pregnant typically have to leave school by will or due to the fear of ostracization by peers and teachers. It is ironic then that once a girl leaves school, sex and marriage are expected [35] and the possibility of returning back to school doesn’t remain. Cultural change in Kenyan context has brought about significant demographic changes (such as adolescent morbidity and mortality) and these changes need to be understood better in their entirety.
  To address these multiple gaps, we adopted an *Engagement Interview* approach to address barriers to care and socioemotional experiences of pregnant adolescents [23, 36]. In using an ‘engagement stance’ of interviewing (further elaborated in the methods section) we have tried to touch on the complex needs of the adolescents i.e., dwelling on health, economic, social and family support, interpersonal, nutritional etc. needs and challenges. Our concern was to understand from the adolescents’ standpoint their mental health distress, challenges, and barriers to receiving care in this vulnerable stage of their life. The key question we asked was: what were their experiences in relation to their care, social support, and interpersonal relationships? Two related questions were 1) how does depression and other mental health issues get noticed and addressed by the caregivers and MCH nurses when they felt down, depressed, or stressed and 2) which practical, interpersonal, and cultural barriers were present before these young girls?

## Methods

### Epistemology

Our study was informed more broadly by a grounded theory method. Grounded theory approach does not presume existence of a theory from the outset but uses the participants’ data to create meaningful categories and theoretical thrust [37]. We embedded an ‘engagement stance’ shown by the interviewer which was meant to elicit experiences around pregnancy, emotional support, and quality of interpersonal relationships. We had a broad framework which provided us with three registers to probe our participants in: *a) practical (*e.g.*, resources, access) b) interpersonal (*e.g.*, support from spouse/partner, parents, health centre), and c) cultural barriers (*e.g.*, stigma from family, community, discrimination in school, clinics). The conceptual foundation of this interview is a combination of ethnographic and motivational style probing designed to identify barriers to uptake of services* [23]*, investment in treatment, and tapping into experiential components around the mental health problem namely, depression.* It specifically identifies participants’ practical constraints such as food insecurity, lack of funds to access healthcare, and help in ascertaining the challenges that will interrupt therapeutic engagement and progress.

As a team, we got beginner’s training in the use of G-IPT (with the exception of NG who is already a trained supervisor of IPT by International Society for IPT) adapted for adolescents in perinatal spectrum in the Kenyan context. We went with the interview with the expectation that our adolescent participants will generate a theory about psychological distress associated with being pregnant at a vulnerable age and that we will learn about the severity and interpersonal challenges around being pregnant and depressed. Whilst we had these registers to probe the adolescents on, we did not start the interview process with assumed categories within these broad registers and expected our interview data to generate themes and sub-themes that would highlight these. See Table 1 which presents the interview domains and Table 2 which presents the interview themes and corresponding case vignettes.

**Table 1:** Sample Characteristics and Interview focus

	Characteristics	Focus of the Interview Discussion
Sample 1 (Pregnant Adolescents) *N* = 12	Eight participants with moderate to severe depression, two of our participants were HIV positive, two participants with experiences of intimate partner abuse, only three were in school remaining dropped out, two participants with neither parental nor partner support available	Identifying interpersonal (depression, relationships, social support), practical (food insecurity, material support and care, finances), cultural (extended family and community social support)
Sample 2 (three Caregivers/one partner), *N* = 4	Two of three mothers themselves in vulnerable financial conditions, no support from their spouses, two of them had babies during their adolescents themselves, one partner we interviewed depended on his own mother for support.	Identifying understanding of the adolescent’s health and psychological well being, looking at their own reactions and situations (interpersonal and social), appraisal of family, social and financial support

**Table 2:** Interview themes and corresponding case vignettes from adolescents who visited health facility for maternity care

Domain, underlying dimension and theme	Vignettes from Interviews
1. *Experience of Pregnancy, acceptance/understanding implications*	
a. Denial of the pregnancy	Tuliendelea kuwa marafiki mpaka wakati nilikosa periods kwa miezi 6, nikajua niko na mimba.Nilimwambia akasema nimekuwa na vijana wengine shuleni. *“We continued as friends until I missed my periods for 6 months, I knew I was pregnant. I told him and he said I have been with other boys in our school.”-*15 year old,9 months pregnant “Wazazi walinifukuza nyumbani ndiyo nikaanza kuishi na marafiki zangu” *“I was sent me away from home that’s why I stay with friends”-* 17 year old commercial sex worker,8 months pregnant living with friends Maisha ya ndoa ni ngumu sana, saa zingine akikasirika na mimi, anasema huyu mtoto sio wake, *Marriage life is so hard, sometimes when he is annoyed with me; he says that the baby is not his..* 18 years old
b. Intention to put up the baby for adoption or ask the mother to raise the baby	Daktari akisema mtoto ako sawa, tungependa akizaa, mtoto achukuliwe na childrens’ home au mtu mwenye anamtaka ndio msichana arudi shule kwa vile siwezi kuwalea. *“In case the doctor says the child is healthy, we would like to give him/her to a children’s home or somebody who needs a child so my daughter can go back to school because I cannot raise them”* - 15 years old, 7 months pregnant Sijui kama kuna watu watanisaidia zaidi ya mama yangu kwa vile huyo kijana hafanyi kazi na anashinda akivuta bangi. Mama naye si mbaya lakini anafanya tu vibarua (Analia). *I don’t know whether others will help me except for my mother because the boy* *does not have a job and he takes Marijuana.* *My mother is not bad but she does odd jobs. (Crying) -*15 years old*,*9 months pregnant Nimegundua ni mimi ndio rafiki yake pekee na sitaki awe peke yake. Sote tuko sawa kama wazazi na mama in-law ananisupport. Makosa imefanyika. Sisi tutatunza mtoto, arudi shuleni. Mimi ni counselor, nasaidia wazazi wengi wanapitia haya, kwa nini nisisaidie mtoto wangu wa kipekee? *I have realized that I am her only friend and I do not want her to be alone. We are all okay with this as parents and my mother-in-law supports me. The mistake has already happened so we are going to take care of the child and she goes back to school. I am a counselor and do this all the time for other parents and why not for my only child?-*15 year old’s mother
c. End of education and need to look for job	Nilikuwa naishi na wazazi uko Kawangware lakini juzi walinifukuza sasa naishi na marafiki zangu tuna tafuta pamoja na kupata chakula. Nilifanya standard 8 lakini sikuenda Form 1 juu ya fees. *I am 17 years old, not married. I was staying with my parents in Kawangware but they recently chased me away now I am staying with my friends hustling together and getting food to eat. I did standard 8 examinations but did not go to Form 1 because of school fees-* 17 years old Niliacha shule standardi 8 ju sikulipiwa pesa za mtihani. Nimekuwa tu home naboeka bila kufanya chochote, kazi za nyumba tu. Shangazi hatupendi vile na hatusomeshi anashughulikia watoto wake tu. Nili patana na huyu boy last year na tunaishi na madhake hapa Kangemi. Juzi ndio niligundua niko na ball. *I dropped out in Standard 8 because the exam fee was not paid. I have been home since, being bored without doing anything but house chores. My aunt does not love us that much because she does not educate us but only her children. I met this boy last year and we are staying with his mother here in Kangemi. Recently, I found out I was pregnant* – 18 year old. Niko na 17 years,niliacha shule nikiwa class 6 ju wazazi hawangeweza kulipa fees. Sasa, leo unaenda tu poa na kesho unafukuzwa, nikaamua tu niache shule. *I am 17 years old, I dropped out of school in class 6. My parents could not raise the fees, so today you go to school without incident but tomorrow you are chased away. I decided to drop-out of school.*
d. I tried to abort but that didn’t work out well	Interviewer: Uja uzito huu ni wa nani na anakusaidia? *Who is responsible for your pregnancy and does he support you?* Interviewee: Ni mmoja wa mabeshte zangu. Nikizaa nitajua tu.Wazazi walinifukuza nyumbani ndiyo nikaanza kuishi na marafiki zangu. Nilijaribu kutoa hii ball mara mbili na ilikataa sasa nitazaa tu. *It is one of my friends. When I give birth I will know. My parents chased me away from home, that’s why I stay with friends. I tried to abort twice but it refused now I will just give birth* Interviewer: Ulijaribu na nini? *What did you try it with?* Interviewee:Juice conk na strong tea vile nilikuwa miezi tatu na nne *Concentrated juice and strong tea when it was 3 and 4 months-* 17 year old 8 months pregnant
e. Poverty impact on their pregnancy – absence of nutritious food	Ni katika hali ya kujitafutia ju home hauwezi pata vitu madame wanaitaji na kulala njaa mara mob. Mimi ndiye msoo kwetu kwa hivyo nikileta ata masisters zangu watakuwa poa. Baba anafanya kazi lakini ni mlevi na mama anaoshea wadosi nguo lakini haitoshi. *It was in the course of hustling because at home I could not get what girls need and we were sleeping hungry all the time. I am the eldest and so if I bring even my sisters will be alright. My father works but he is a drunkard and my mother washes clothes for rich people but it’s not enough* Stress kubwa ni kukosa do, inabidi madhake atupe kila kitu na siku hizi naona inaleza kelele sana- 17 years old 8 months pregnant *The biggest stress is lack of money. His mother has to provide everything and these days I see it causing a lot of noise-* 18 year old married. Babu ndiye kama baba saizi ju baba yetu alituacha. Tuko watoto sita kwa familia. Kwa wakati huu, sijanunua chochote cha mtoto. Sina pesa. *My Grandfather is like our father now because my father left us. We are six siblings. Upto this moment, I have not bought anything for the baby. I don’t have money* - 15 years old 9 months pregnant
2. *Attitude of and support from the boyfriend*	
a. Denial of pregnancy	Mimi na huyu kijana tumekuwa marafiki kwa miaka mmoja na miezi saba. Nilifikiria huyu kijana ndiye rafiki yangu na ananipenda.nikakubali kuwa naye……. Nimepata wakati mgumu sana kwa vile nilikuwa niko class 8 najitayarisha kufanya mtihani. Nilificha kwa miezi tano lakini ikaanza kuonekana. Kijana pia amekataa huyu mtoto. *We have been friends for 1 year and 7 months. I thought the man was my friend and loved me that’s why I accepted to be with him……………I have had a very hard time since I was in class 8 preparing for my exams. I hid it for 5 months but it started to show. The boy has denied the child.- 15 year old* *7 months pregnant* Tulikuwa tumezoeana kama majirani. Siku moja nilienda kumtembelea akanishawishi tufanye hiyo maneno na nikakubali. Tuliendelea kuwa marafiki mpaka wakati nilikosa periods kwa miezi 6, nikajua niko na mimba.Nilimwambia akasema nimekuwa na vijana wengine shuleni. *We used to get along as neighbours. One day I went to visit him, he convinced me to do that thing and I agreed. We continued as friends until I missed my periods for 6 months, I knew I was pregnant. I told him and he said I have been with other boys in our school-* 15 year old, 9 months pregnant
b. Even the boy’s family denies any responsibility	*I am thinking that when she gives birth, I will take the child to his/her father,* *leave him/her in their doorstep. I do not have a job. I am dependent on my husband.* *We have 3 children; the last 2 were twins 10 months old. It’s been hard living with her* *and this pregnancy. I cannot take care of another child-* 15 year old’s mother, 7 months pregnant *I wrote a letter and took to their home, his mother was also shocked,. In the letter* *I told them as the mother, I will inform you of her progress, but I would also* *like you to talk to your son so we all see the way forward. I wrote the letter* *because I didn’t want to talk too much out of anger. So far I have not got any positive feedback. My husband does not want me to call them.-*15 year old’s mother, 6 months pregnant
c. He is very supportive and trying his best	*He supports me a lot so I see that I will be fine* – 18 year old married *He is a friend of mine since primary school, since I got pregnant, he has not given me stress, he has accepted and even come to speak to my parents- 17 year old* *I have known my husband for a while and when I told him that I am pregnant, he said we stay together and be a family – 17 year old*
3. Attitude and support from mothers and significant caregivers	
a. Mother is quite angry and disappointed	*Interviewer*: Mara ya kwanza kusikia hii maneno, ulijiskia aje? *The first time you heard about it, how did you feel?* *Interviewee*: Imenikera sana*. It has annoyed me very much –* 15 year olds mother(7 months pregnant) Msichana wangu ni mmoja tu,nimemlea kanisani, hata sijui nini ilifanyika? *My daughter is my only girl, I have brought her up in the church, I just do not know what happened – 15 year old’s mother(6 months pregnant)*
b. Lack of family support	Nilikuwa naishi na nyanya na shangazi zangu kule ushago kwa sababu mama alioewa hapa Nairobi. Walikasirika sana na wakanirudisha kwa mama yangu, *I used to live with my grandmother and aunties in rural home because my mother is married here in Nairobi……….My grandmother and aunties were very annoyed with me and told my mother-* 15 year old-7 months pregnant Babu alisema anakuja kunichapa kwa vile nimehaibisha familia. Yeye ndiye kama baba saizi ju baba yetu alituacha. Tuko watoto sita kwa familia…… Sijui kama kuna watu watanisaidia zaidi ya mama yangu kwa vile huyo kijana hafanyi kazi na anashinda akivuta bangi. Mama naye si mbaya lakini anafanya tu vibarua *My grandfather said he will come to Nairobi and beat me up because I have embarrassed the family. He (grandfather) is like our father now because my father left us. We are six siblings…….. I don’t know whether others will help me except for my mother because the boy does not have a job and he takes Marijuana. My mother is not bad but she does odd jobs. –* 15 year old 9 months pregnant. *“I am 17 years old, not married. I was staying with my parents in Kawangware but they chased me away now I am staying with my friends”* Nikituma pesa nyumbani pia anakasirika na mimi. Nikichelewa kutoka kazini anasema niko na vijana wengine huko nje. Mdosi aliniambia nikifanya kazi vizuri, ataniongeza pesa. Nafikiria kukomboa nyumba yangu lakini wazazi nyumbani pia wananitegemea nitume pesa *If I send money home, he also gets annoyed with me. If I am late from work, he says I am with other men outside. My boss says if I work well, she will give me a raise. I am thinking of renting a house but my parents at home rely on me to send them money-* 18 year old *I understand that my parents are upset but see it as my first big mistake. I have been a good student all through……..so I don’t know why my parents, especially dad is still upset- 17 year old just cleared Form 4*
c. Get support from social and community health workers	*Interviewer:* Unadhani utaweza kulea mtoto pekee yako? *How do you think you will manage to raise the child on your own?* *Interviewee:* Mimi ni volunteer social worker Napata huku na kule na niko na savings sio mingi lakini nitamanage. *I volunteer as a social worker and have some savings from a previous job and I think it may not be enough but I can manage on my own* *Interviewer:* Unajihisi aje juu ya huyu mtoto? *How do you feel about your baby?* *Interviewee:* Napenda mtoto wangu sana na natamani kumuona. Babake ndio sitaki. I *love my child and long to see him/her. It’s the father that I do not want* *Interviewer:* Hiyo ni vizuri, unaishi peke yako kwa sasa? *Are you staying alone now?* *Interviewee:* Ndiyo naishi peke yangu *Yes I am staying alone* *Interviewer:* Je, unapata support? *Do you get social support currently?* *Interviewee*: Ndiyo, napata support kutoka kwa marafiki na social workers wezangu kama Lilian. *“I get support from my friends, social workers here at the hospital like Lilian”-* 18 year old
4. *Other challenges impacting depression*	
a. Experiencing domestic violence	*Marriage life is so hard, sometimes when he is annoyed with me; he says that the baby* *is not his. When I talk to neighbours, he gets annoyed and beats me.* *My husband said I use his name and not my father’s name*. *If I send money home, he also gets annoyed with me. If I am late from work, he says I am with other men outside. My boss says if I work well, she will give me a raise. I am thinking of renting a house but my parents at home rely on me to* *send them money –* 18 year old married,5 months pregnant employed *No, he slaps me on the cheeks-* 18 year old 9 months pregnant
(b) Substance use - consuming Miraa (Khat)everyday	*It’s normal where I come from and it doesn’t affect you at all……. Me and my husband* *sell it, he gets from home and I can take it anytime I want* Interviewer*:- I would just like to advise you that Khat is a stimulant just like other drugs and it can affect the health of the child. Would you consider stopping for the duration of the pregnancy?* *I can try but I know it doesn’t affect because people in my area take it every day.* *−* 16 year old married
*Plans for the future*	
a. Will have to plan a life independently	*I volunteer as a social worker and have some savings from a previous job and I think it may not be enough but I can manage on my own…..I don’t see him thinking ahead and preparing for the future life together-18 year old social worker*
b. Parents/partner plan to send me back to school	Makosa imefanyika. Sisi tutatunza mtoto, arudi shuleni *The mistake has already happened so we are going to take care of the child and she goes back to school-* 15 year old’s mother Mimi pia nataka kupeana mtoto kwa vile siwezi mulea na nataka nirudi shule. *I would also like to give up my child because I cannot take care of it and I also want to go back to school-* 15 year old Interviewer*:“Did you want to continue with school but you were unable to that’s why you went to get married?”* Adolescent*: “Yes, he had promised to take me to school after delivery”* *-* 16 year old
c. We live on meagre resources so don’t know how we will look after the baby	*“I do not have a job. I am dependent on my husband. We have 3 children; the last 2 were twins 10 months old. It’s been hard living with her and this pregnancy. I cannot take care of another child”–* 15 year old’s mother

### Setting

We worked in one of the Nairobi county health center that is typically headed by a nursing officer-in-charge. The facility offers community level care services to include maternal and child health services, HIV counselling and testing, and anti-retroviral therapy. A small cubicle was provided by the health facility to conduct these interviews.

### Participants and recruitment process

This study is part of the larger initiate of prenatal depression project (which aims to study prevalence of depression and mechanisms for depression in pregnant adolescents). We were given IRB clearance from Kenyatta National Hospital and University of Nairobi Ethics Review Committee (ERC Approval No. P499/07/2015).

We carried out data collection after receiving authorization from Nairobi County Directorate. In the larger study, 174 pregnant adolescents were recruited, 32.9% were identified with depression (using PHQ-9 cut off of 15 and above) [38]. We used a convenience sample to recruit 12 out of the larger cohort we had interviewed for our cross-sectional survey study. We made a decision to recruit a sample of 12 participants who screened positive for depression with at least 5 participants with severe depression symptoms on PHQ-9 and the remaining participants (these 7 participants scored mild to moderate depression severity on PHQ-9) were identified through their responses to the sociodemographic questionnaire which sought to identify 5 domains of psychosocial risk factors namely, lack of perceived social support, living with HIV/AIDS, domestic and sexual violence, substance abuse, and poverty. Two participants declined to give consent for in-depth interviews due to reluctance expressed by their family member who had accompanied them*.* All the adolescents were provided referrals (to KNH Department of Mental Health or to the Psychiatry clinic that runs in the health center in cooperation with the University of Nairobi) after the interview. See Table 1 for more information. We interviewed 3 caregivers and 1 partner who accompanied the adolescent for check-up to get a sense of the family’s experiences and perspective around the developing pregnancy. Several of our participants mentioned not having enough monetary support for a caregiver, partner or a boyfriend to accompany so we were only able to interview a few caregivers we could manage to find in the clinic.

Informed by grounded theory and engagement interview approach, we developed an interview guide for the adolescents and a separate guide for caregivers and partners (see Additional files 1 and 2). The study entailed questions of sensitive nature, and it was imperative to protect participants and their caregivers’ identities.

### Data collection

We interviewed the 12 adolescents separately in an in-depth semi-structured interview format. Sample 1 (pregnant adolescents) and 2 (caregivers/partner) interviews were both key informant interviews and therefore done on a one-on-one basis. We used the interviews without identifiers for adolescents (Additional file 1) and for caregivers and partners (Additional files 2) to elicit experiences of pregnancy, depression, and barriers to care.

The interview was carried out by a trained researcher who was the first author in this case. Consent form was signed by participants prior to the interview. Interviews were carried out in Kiswahili or English based on the preference of the participants and using the procedure that was sensitive to participants’ level of distress (e.g., provided additional referrals or social support as needed after individual interview). The same procedure was followed with the 4 caregivers: 3 mothers and 1 partner and the researcher gave time to address any questions the adolescents and caregivers had about mental health issues.

### Data analysis

Audio tape data from interviews were transcribed and then analyzed by a team of 3 individuals. The first author read each transcript and developed themes per interview. The second author who is a native Kiswahili speaker reviewed the interviews to check quality of translation and transcription. These interviews and the preliminary themes were then reviewed by second and last authors who were the primary researchers’ supervisors. This team then shared key interview vignettes, themes, and codes to other members of the team for their feedback and deliberation. Two sample interviews were then read by the other members of the team. The first and last author then triangulated and collated interview themes from these deliberations and these were then categorized into core themes. Key themes and the corresponding codes and relevant vignettes from all transcripts were compiled in to MS word documents by the primary researcher and reassessed by the co-authors for accuracy, authenticity, and synergistic links with our epidemiological framework. We have tried in our analysis to keep closer to our participants’ lived experience and presented their vignettes to exemplify their feelings and thoughts. Table 3 elucidates the core themes and presents representative vignettes on the right column. The table’s first section is devoted to the adolescent participants and the second section focuses on the 4 caregivers we interviewed.

**Table 3:** Emerging Themes and illustrative quotes on challenges experienced by caregivers living with adolescents

Domain, underlying dimension and theme	Case Vignettes
1. Adolescent Pregnancy: Acceptance and Implications	
(a) Acceptance of the pregnancy	*I have been counseled by the school and I have accepted my daughter’s condition* *Interviewer:* Na baba yake? *What about the father?* *Mother:* Alikasirika wa kwanza lakini nilimwongelesha na ako sawa kwa sasa. *He was angry initially but I have talked to him and he is ok now-*15 year old’s mother
(b) Worries about the birth of the baby	*“In case the doctor says the child is healthy, we would like to give him/her to a children’s home or somebody who needs a child so my daughter can go back to school because I cannot raise them” = 15 year olds mother* *The mistake has already happened so we are going to take care of the child and she goes back to school-*15 year old’s mother *“I am thinking that when she gives birth, I will take the child to his/her father, leave him/her in their doorstep”-* 15 year old’s mother
(c) Self-Blaming for the circumstances the daughter is in	*My daughter is my only girl, I have brought her up in the church,* *I just do not know what happened. -* 15 year old’s mother *She is the one I gave birth to when I dropped out of school.* *Her father and grandmother rejected me until I gave birth to her and she* *resembled her father so much. I left her with my mother when* *I got married here in Nairobi. Perhaps my past is catching up with me.* *I mean would my daughter should go through the same experiences* *as me if it’s not fate-*15 year old’s mother
2. Attitude of fathers and extended family	
Possibility of shame, isolation and lack of commitment by fathers within the family	“*I do not have a job. I am dependent on my husband. We have 3 children;* *the last 2 were twins 10 months old. It’s been hard living with her and this pregnancy.* *I cannot take care of another child. My husband is silent on this matter* *but I know he is not happy” My mother has returned my burden to me-* 15 year old’s mother with reference to the daughter living with her and her step-father as opposed to the grandmother *“He was angry initially but I have talked to him and he is ok now”-* 15 year old mother
3. Plans for the future (Responses from Spouse and Mother)	
(a) Seek economic sustainability(Spouse	Interviewer to spouse:-Umepanga aje? *What are your plans?* Spouse: Nimeanza hii job ya boda boda juzi, ikikua poa, tutakuwa na pesa za kujitegemea si wenyewe *I recently started a boda boda business, if it goes well, we will have* *money to rely on ourselves-* 23 year old spouse
(b) Seek financial assistance (Mother)	Mama alisema ataomba dada yangu mkubwa mwenye anafanya kazi ya nyumba uko Mombasa kutusaidia *My mother said she will ask my sister who is a house help in Mombasa to help us.* - 15 year old 9 months pregnant *Interviewer:* na mwenye anahusika? *What about the partner concerned*? *Mother:* Ni mtoto kama yeye. *He is also a child like her* *Interviewer:* Na wazazi wake? Munajadiliana? *What about the parents, have you had any discussion with them?* *Mother:* Niliandika barua nikapeleka kwao, mama yake alishtuka. Kwa barua, niliwaambia kama mama yake, nitawaeleza vile anaendelea lakini ningependa uongee na kijana yako tuone way forward. Niliandika barua kwa sababu sikutaka kuongea sana juu ya hasira. Mpaka wakati huu, hatujapata feedback. Bwana yangu hataki niwapigie simu. *I wrote a letter and took to their home, his mother was also shocked,. In the letter I told them as the mother, I will inform you of her progress, but I would also like you to talk to your son so we all see the way forward. I wrote the letter because I didn’t want to talk too much out of anger. So far I have not got any positive feedback. My husband does not want me to call them.* 15 year old’s mother

### Methodological and ethical considerations

The parents and/or guardians of the minors in this study provided informed consent to participate. We would like to highlight a few caveats that underpin our experiences of engaging with the pregnant adolescents:

- *High risk participants:* Food insecurity and basic financial problems were rampant in addition to the challenges around receiving social acceptance and access to medical care. Two of our participants were HIV positive and 1 of them was a commercial sex worker entirely on her own in a predatory environment. Out of 12 participants interviewed, 5 participants were strongly advised to go to the psychiatric clinic or to the youth center at Kenyatta National Teaching and Referral Hospital for immediate follow-up care and remaining were referred to the mental health clinic at the facility. We made the nurse-in-charge aware of these referrals.
- *Experiential understanding of depression and various barriers*: In our depression prevalence and psychosocial risk study [38] we found that 58% (out of 174 participants) scored positive for likelihood for antenatal depression using EPDS and about 15.9% (*N* = 28) had severe depression symptoms on PHQ-9 scores of 15 and above. We felt that the grounded theory approach infused with ‘engagement’ related barriers appraisal was a helpful strategy to understand their distress fully.
- *Multi-disciplinary team of researchers:* This qualitative inquiry was greatly facilitated by our team of diverse researchers comprising of a psychiatrist (PK), two clinical psychologists (JO and MK) and others with expertise in developmental psychology and clinical social work including treatment of perinatal depression (NG) and child and adolescent mental health interface with implementation science (KYH). This added a holistic perspective in drawing key perinatal adolescent mental health challenges, understanding ethical issues underpinning pregnancy in adolescence, and adolescent reproductive and sexual health policy implications. This array of backgrounds shows the diversity of perspective on the issue.

## Results

The qualitative results were categorized in 4 major themes including depression, anxiety and stress around the pregnancy, denial of the pregnancy, lack of basic needs provisions and care, and restricted opportunities for personal development post pregnancy.

- *Depression, anxiety, and stress around the pregnancy:* Once the pregnancy status was known our participants shared that they *experienced low mood, anxiety, and stress* in one way or another as illustrated below:
*Interviewer: Please tell me why you are crying.*
*Adolescent: It’s painful*
*Interviewer: What kind of pain?*
*Adolescent: That of offending my parents*
  *“Adolescent’s Mother (intervenes): Right now, she sleeps a lot and does not eat, she is always indoors”- *(15-year old’s mother).

Our participants recounted times when they were overtaken with feelings of shame, anxiety, and fear; they felt like denying the pregnancy even when the signs were obvious such as missing periods for several months. Another 15-year old expressed her feelings as follows:

*“I feel embarrassed all the time and also feel that I brought shame to them”.*

b) *Denial of the pregnancy:* This was not only a lived experience of the pregnant adolescent, but the same applied to their partners and her family who felt very concerned about their reputation in the community. Another 15-year old recalled her partner’s reaction below.
*“We have been friends for 1 year and 7 months. I thought the man was my friend and loved me that’s why I accepted to be with him . . . I have had a very hard time since I was in class 8 preparing for my exams. I hid it for 5 months but it has started to show. The boy has denied the child.” – 15-year old, 7 months pregnant*
  *“We continued as friends until I missed my periods for 6 months, I knew I was pregnant. I told him and he said I have been with other boys from our school.” –* 15-year old, nearing 9 months.

This situation is compounded by social stigma from the immediate and extended family who feel that the pregnant adolescent has brought shame and disgrace to the family. A vignette here captures this low mood.

*“I was sent away from home when I got pregnant that’s why I stayed with friends.” –* 17-year old commercial sex worker, 8 months pregnant

*“I don’t know whether I am depressed the way you describe it but I regret a lot . . . (crying again). I went to a bridge at home and wanted to throw myself because my grandfather is very harsh and upset with me and an aunt told me it is a sin.” –* 15-year old, 9 months pregnant.

In both vignettes, the immediate practical situation of not having any support or being ostracized by family seems to mask depression. Depression manifestation is in terms of immediate worries and steps taken for survival.

c) *Lack of basic needs provisions and care:* It emerged that one of the major precipitating factors for depression in some of the participants was lack of basic needs such as food, decent shelter, and clothing which the pregnant adolescent anticipated would directly and negatively impact on the welfare of the unborn child. Most of the caregivers felt the burden of sustaining their present economic challenges would be compounded by the birth of an extra mouth to feed as described below:

*“I am thinking that when she gives birth, I will take the child to his/her father, leave him/her in their doorstep. I do not have a job. I am dependent on my husband. We have 3 children; the last two are twins 10 months old. It’s been hard living with her and this pregnancy. I cannot take care of another child.” –* 15-year old’s mother (adolescent daughter herself is 7 months pregnant).

One of our participants mentioned that her mother had thought about putting the baby for adoption and was hoping that the baby would be delivered healthy so it could be passed on to a family who would look after it.

*“In case the doctor says the child is healthy, we would like to give him/her to a children’s home or somebody who needs a child so my daughter can go back to school because I cannot raise them.”* – 15-year old, 7 months pregnant

The experience of one of our participants who engages in commercial sex for a living was very telling and we learnt how families disenfranchise adolescents without providing any care or solutions during such situations.

*“I got pregnant in the course of hustling because at home I could not get what girls need and we were sleeping hungry all the time. I am the eldest and so if I bring even my sisters will be alright. My father works but he is a drunkard and my mother washes clothes for more affluent people but it’s not enough…” –* 17-year old, 8 months pregnant.

d) Restricted opportunities for personal development post pregnancy: Most of the participants were from low economic backgrounds and expressed regret due to the extra burden to their caregivers, imminent termination of their education, and the need to have to be gainfully employed to care for their unborn children. A 15-year old narrated the following:
  *“My grandfather is like our father now because my father left us. We are six siblings. Up to this moment, I have not bought anything for the baby. I don’t have money… I don’t know whether others will help me except for my mother because the boy does not have a job and he takes Marijuana. My mother is not bad but she only does odd jobs.” –* 15-year old, 9 months pregnant

*“I was staying with my parents in Kawangware but they recently chased me away now I am staying with my friends hustling together and getting food to eat. I did standard 8 examinations but did not go to Form 1 because of lack of school fees.” –* 17-year old, 8 months pregnant

However, one mother recounted the following in a reconciliatory and heartening tone:

*“I have realized that I am her only friend and I do not want her to be alone. We are all okay with this as parents and my mother-in-law supports me. The mistake has already happened so we are going to take care of the child and she goes back to school. I am a counselor and do this all the time for other parents and why not for my only child?” –* 15-year old’s mother

This optimistic stance of a caregiver was not shared across the board. For most of our participants pregnancy denoted an end of education and along with that came the pressure to contribute to resources including money to look after the additional member of the family.

“*I do not have hope. I have many thoughts because we do not have money and I do not like insults and noise from his mother. I do not sleep well, eating is tough sometimes but he is trying.*” – 17-year old married, 4 months pregnant and living with her mother-in-law.

This adolescent was coping with a new relationship with her husband’s mother (her mother-in-law) and whilst we do know the mother-in-law’s own story or resources, the adolescent’s vignette makes it clear that the pregnancy has added to the familial stress.

Similar experiences were recounted with our participants sharing that even when they managed to go to school, the paucity of funds kept coming in the way and the school would threaten to discontinue them forcing them to save grace and drop out on their own.

*“I dropped out of school in class 6. My parents could not raise the fees, so today you go to school without incident but tomorrow you are chased away. I decided to drop-out of school.” –* 17-year old, 7 months pregnant

The most potent theme interspersed along this experience of dejection, sadness and physical adversity (in the form of pregnancy) was of poverty and continuous financial and food insecurity. A vignette from one of the participants underscores the churning and torment adolescents go through in a resource-constrained household, sharing burdens, and forced to become adults early in their lives.

We were particularly interested in the role of the male partner or husband and we got a very grim picture where the male support recedes into darkness once an adolescent becomes pregnant. This takes the form of denial of the role and responsibility of the male partner/friend in contributing to the pregnancy and later during involvement of the family this is repeated with the partner’s family that refuses to provide any support. The family dynamics and the tussle varied from one participant to the next but there was a long process of engaging with a distant and potentially hostile male partner.

An interview vignette from a participant’s mother is very telling about the lone struggle in this matter.

*“I wrote a letter and took to their home, his mother was also shocked. In the letter I told them as the mother, I will inform you of her progress, but I would also like you to talk to your son so we all see the way forward. I wrote the letter because I didn’t want to talk too much out of anger. So far I have not got any positive feedback. My husband does not want me to call them.” –* 15-year old’s mother, 6 months pregnant

Whilst for 7 of our participants, partner support was entirely absent; there were 5 participants (1 of whom was accompanied by the partner to the clinic) who articulated support and care that was given. We found that 4 of these adolescents were married and this helped in garnering support as the relationship was ‘formalized’. One of them shared this heartening example of her experience.

*“He is a friend of mine since primary school, since I got pregnant, he has not given me stress, and he has accepted and even come to speak to my parents.” – 17-year old*

Our participants made us understand that the support of the family particularly the parents and mother especially is critical for the long-term future of the adolescent. We also learnt that this support cannot be taken for granted and comes in stages of negotiation and balancing anger, stigma and hurt with feelings of care, and sympathy for the adolescent. Feelings of dejection and sadness overwhelmed the mothers we interviewed as they felt that they had failed as a parent. Often times it is the mother who is blamed for the fate of the adolescent. One of the mothers we interviewed recounted in a distressed tone:

*“My daughter is my only girl, I have brought her up in the church, I just do not know what happened.” – 15-year old’s mother (daughter was 6 months pregnant)*

The sadness often had tones of anger and worry for the adolescent. However, despite this outburst our adolescent participants found their mothers to be the most receptive of the caregivers and there was some hope that she would be angry but support the daughter. The mother and daughter often had to team up to bear the burden of the pregnancy for the family and social ostracization that ensued. A vignette from one of our participants is very telling in this regard.

*“I used to live with my grandmother and aunties in rural home because my mother is married here in Nairobi … My grandmother and aunties were very annoyed with me and told my mother.” –* 15-year old, 7 months pregnant

The experiences of being thrown out of the house by angry parents and an example of an adolescent participant being threatened by her grandfather that he would come from rural home and beat her. This severely worried her as the father had left and the grandfather took charge of the family. The family’s future was also threatened by the pregnancy and the young girl was aware of that.

Another married participant in a distressed tone mentioned that the partner doubted her and questioned whether the baby was his and she feels lack of freedom and respect in the relationship. This 18-year old participant, who was close to full term, shared some troubling experiences of domestic violence. Her partner would beat her, smacking her on her cheeks and doubting her fidelity and he appeared to control her finances. Her parents depended on her income but that was also a source of rancor between the two.

She was referred to the GBV services and advised to seek support from the mental health specialist visiting the health facility. Some of our participants did acknowledge that the unexpected pregnancy had toppled the balance within their own family, throwing the parents off guard and making them upset. They hoped that their naiveté and the potential to work hard in school and at home would be noticed and that the family would rally support around them.

One of our participants lived alone and mentioned that she had some savings from a job. She had volunteered as a social worker previously so had friends who would help and she would use the health facility affiliated with the social and community health workers to get help with delivery and supplies.

Amongst other issues that complicated the experience of pregnancy was the fact that there was a positive diagnosis of HIV (in two of our participants). One of them worked as a commercial sex worker and her parents had severed ties with her. The other participant was on ARVs and had support from her partner but her own parents were deceased. The couple lived in dire poverty and resource constraints. Another one of our 16-year old participant was addicted to Miraa^1^ which she continued chewing despite being 5 months pregnant. When the interviewer expressed concern, she said that it was widely chewed where she lived and didn’t think it would cause any harm.

In the interviews with the caregivers, namely the 3 mothers we felt there were mixed emotions expressed. Sometimes the health workers and at other times the school counsellor helped prepare the mother to accept the daughter’s pregnancy and to start thinking about the next steps. In a very poignant vignette, mother of a 15-year old recounts her experience.

*“She is the one I gave birth to when I dropped out of school. Her father (mother’s partner) and grandmother initially rejected me until I gave birth to her and she resembled her father so much. I left her with my mother when I got married here in Nairobi. Perhaps my past is catching up with me.” –* mother of a 15-year old adolescent

Two of our participants’ mothers alluded to the stressful deliberations around resources that would take place with their husbands. If the husband was a step-parent to the adolescent, the nature of family dynamics would change as she would be asked to look after the needs of the pregnant girl on her own.

We interviewed one spouse who accompanied his 4 months pregnant wife. He was himself 23 years old and depended on his mother for support. It was reassuring to see that the mother-in-law had accepted the pregnant adolescent and was providing for the couple but there were misunderstandings on finances and pressure to find work and to attend to the increasing needs which troubled the young man.

## Discussion

Results presented above have highlighted the key themes namely *depression, anxiety and stress around the pregnancy, denial of the pregnancy*, *lack of basic provisioning and care, and restricted opportunities for personal development post pregnancy*. Using the engagement framework, which sought to highlight challenges in practical, interpersonal, and cultural domains, we can map these themes onto more concrete barriers that restrict these vulnerable girls’ socioemotional development and future opportunities.

The unique contribution of our study is that it highlights specific areas using the engagement interview format where these vulnerable adolescents need focused psychosocial support. It also helps to identify domains where individual versus family/interpersonal interventions techniques could be used in addressing depression. Within the broad framework of identifying depression risk factors qualitatively, our study identifies stress, anxiety, and fear of the future as themes that dominate the clinical presentation of depression. Depression is known by persistent low mood, sadness, inability to be active or participate meaningfully in daily activities and reduces cognitive, interpersonal, and social functioning. Given the antenatal context, sleep and appetite are also affected. The domains we have identified in the select participants enables us to review the impairments in a finer granular analysis.

### Challenges in practical/resource domain

#### Lack of basic provisioning and care

Pregnancies in adolescents from resource constraint context pose greater challenges, especially around financial access, moral and material support, interpersonal relationship within families and with partners, and stigmatization by health workers when seeking health care. These challenges may further lead to perinatal depression and exacerbate adverse outcomes for mother and child and for communities in perpetuating the cycle of poverty. Poverty serves as both an antecedent and consequence of adolescents pregnancy. Lack of food, poor housing, school drop-out, and compulsion to engage in income generating activities, all increase their vulnerability to transactional sex, early marriage, sexual experimentation, unintended pregnancies and STI/HIV, and continued poverty [23]. Our participants vociferously articulated these themes as they shared the challenges of being pregnant and feeling vulnerable and unsure of how to plan a secure future. Wanting to plan the future independent of the partner or family member appeared as the most obvious choice before them. Two of the participants expressed willingness to return to school if their mothers would look after the baby (something which the family was discussing). Similar themes emerged based on caregivers/partners’ interview data, which also found caring for the pregnant adolescent was a challenge given the family resources. There were also concerns about meeting the new born baby and the adolescent mother’s future needs. Two of the mothers mentioned that the unmarried adolescents’ growing family often clashed with the mother’s own leading to high food insecurity and increased paucity of resources.

### Challenges in interpersonal domain

#### Lack of emotional support and oppressive familial dynamics

Another compelling challenge was the interlocking of the fate of the adolescents’ mother and the adolescent herself if it were a two-parent household. The mother of the adolescent had to persuade and garner support from her husband/partner and fight to allocate resources towards the delivery and baby’s care. In this process, our participants including the mothers we interviewed described how arduous this process is: full of stigma, humiliation for the mother, and being held responsible for the adolescents ‘transgressions’. The mother’s own marriage and relationships undergo turmoil that sometimes yields very negative outcomes (prolonged verbal and physical abuse, abandonment). The culture of female shaming and female blaming strongly influences the outcomes of household bargaining [39].

*Presence of social stigma.* Reviewing the experiences that our participants shared we were compelled to think of the role of men in the entire sequel of the adolescent getting pregnant to the birth of her infant. Six participants from our study cohort lived with partners. Seven adolescents shared the absence of support from their spouse/partners, 4 of whom were abandoned by their partners once the news of pregnancy is known. Some of these are older men who take advantage of the young, naïve girls and others are young men (often adolescent themselves) who become afraid and confused running away from any ownership or accountability. The father of the adolescent herself in most parts came across as a punitive and angry figure whose support and goodwill could not be taken for granted.

### Challenges from the socio-cultural domain

#### Restricted opportunities post pregnancy and selective investment in their care

In the Kenyan context (true for African cultural context as such), a bright adolescent who fares well in education and has come to secondary education would be preferred for possible family intervention in the case of an unexpected pregnancy. However, if the adolescent had mental health or physical or other adjustment challenges at home or school, they are not likely be offered the opportunity to return to school or offered greater social/family protection. Thus, the pregnancy throws the adolescent in a high risk vulnerable context with regards to the provision of the meagre resources that is shared with her offspring [28].

Our study findings underscore that both *structural issues* and *culture* impact outcomes. Our inquiry exemplifies that financial and interpersonal challenges are causes and consequences of perinatal depression [29, 40]. Unexpected pregnancy, rejection from the male partner and family, reluctant family support and resources, continuous food insecurity and poor health care epitomized the experiences of depression and adversities that the adolescent participants shared in this inquiry. While the qualitative nature of our inquiry prevents us from drawing broad generalizations about the adolescents’ pregnancy and their accompanying socioemotional experiences, our findings do concur with the recent qualitative studies carried out in SSA regions of Uganda [14,], and Ghana [41].

## Conclusions

We have highlighted the various social determinants that impact pregnant adolescents. Our participants have shared the nature of their distress, crushed hopes, aspirations as well as limited opportunities as they became pregnant. We also came to appreciate that the adolescent became entirely alone with the exception of some support from her mother or partner. The engagement stance adopted in our interviews elicited the practical, interpersonal, and cultural barriers to uptake of care and factors that negatively impact adolescent mood and well-being. We feel the following next steps are critical in strengthening adolescent health via educational and reproductive health programming and making health and community services friendly and responsive to adolescents.

### Presentation of ‘idioms’ of depression

Depression in our sample presented itself as a combination of low mood accompanied by extreme worries and stress. In a resource-constrained context where there the pregnant adolescent has poor social support, these girls are gripped and mostly preoccupied by practical and cultural constraints. Enhancing the quality of life, access to health care, education, and mental health services are not their priorities. In such vulnerable groups we suspect that the features of depression could become more potent and clearer as these practical and cultural constraints cease to exist. Any depression intervention for such a group has to treat co-morbidities such as trauma, stress, and anxiety alongside with stigma, discrimination, and self-blame.

### Strengthening sexual and reproductive health education

A review study on parent-child communication about sexuality and HIV puts into context lived experiences of a majority of our participants [42]. The review findings indicated that parental discussions tend to be authoritarian and uni-directional, characterized by vague warnings rather than direct, open discussion in addition to young people encountering several barriers in open and friendly communication. The greatest injustice to children and young people takes place in their own households and [33] this is also true in the context of pregnant adolescents. Though we did not explore if the adolescent had knowledge about contraception, safe, and consensual sex; it was clear that the level of awareness of participants in our study was limited [34]. One of the adolescents mentioned that ‘*she was trying to abort her baby by drinking more black tea and concentrated juice.’* Developing an educational response to assist in combating adolescent pregnancies is critically needed. Increasing engagement of parents and teachers at the school and health facility levels are crucial for both adolescent boys and girls.

### Training health workers in identifying mental health problems in high risk adolescents

Developing innovative and scalable interventions to reduce the adverse consequences of depression in HIV positive pregnant adolescents and support their engagement in the health care system is another important area [43]. These innovative interventions have to factor in various barriers and all possible registers in which the pregnant adolescents struggle to develop a future [44]. In another paper [45], we have provided a multi-stakeholder perspective into adolescent pregnancy and associated mental health challenges including depression and it is only when we carefully understand perspectives of relevant stakeholders can the health services delivery improve.

### Implementation of adolescent friendly services at community, school and health facility

Innovative practices such as cash transfers that address food insecurity, basic material provisions for adolescents offer unique solutions [46]. Handa et al., [47] describe a cluster randomized trial using unconditional cash transfers to make direct impact on adolescent pregnancy through increasing the enrollment of young women in school, financial stability of the household and delaying age at first sex. Another innovative multi-tiered intervention by Austrian et. al. [48] with 6000 adolescents ages 11–14 in Kibera and Wajir regions focusing on violence prevention, educational, health, and wealth generation interventions all nested together aims at addressing the multidimensional needs of the adolescents. It has been suggested that incentive-based interventions could reduce the burden of adolescent pregnancies and keep girls in secondary education [49]. Akin to the Austrian et. al. trial, in another recent study on 452 married adolescents from Kenyan urban slum settlements, Muthengi et al., [50] found that compared to girls who did not work, working with no regular savings was significantly associated with greater odds of experiencing IPV whereas, asset building and life skills development enables the adolescent girls to lessen dependency on men and therefore be less exposed to violence too. Education therefore is one of the key effective strategies to improve girls’ self-worth and health, and long-term productivity and it needs to be strengthened with effective policy stipulations on ground. Our findings underscore the need to bridge the implementation gaps in offering adolescent friendly services at community, primary care, and educational contexts.

## Additional files

Additional file 1: Interview guide with pregnant adolescents (DOCX 27 kb)

Additional file 2: Interview guide with caregivers and partner (DOCX 22 kb)

## Abbreviations

CHWs: Community Health workers
IPV: Intimate partner violence
KES: Kenyan Shillings
KHDS: Kenya Health Demographic Survey
KNH: Kenyatta National Hospital
NASCOP: National AIDS and STIs Control Programme
PHQ-9: Patient Health Questionnaire- 9
UNFPA: United Nations Population Fund
UNICEF: United Nations Children’s Fund
WHO: World health organization

## Footnotes

1 Miraa is also known as Khat, is a psychoactive substance and classified as an amphetamine like stimulant and is endemic to the Horn of Africa. The leaves of the plant are chewed and it has several short and long term damaging effects such as euphoria, increased heart rate, hyperactivity and psychosis.
