# Supplementary material for: Adversities and mental health needs of pregnant adolescents in Kenya: identifying interpersonal, practical, and cultural barriers to care
Source: BMC Womens Health. 2018 Jun 15;18:96. doi: 10.1186/s12905-018-0581-5 (PMC6003032; doi:10.1186/s12905-018-0581-5)
Supplement: Supplementary file 1 — Interview guide with pregnant adolescents (DOCX 27 kb) [file 12905_2018_581_MOESM1_ESM.docx]

**Additional File 1 IN-DEPTH INTERVIEW GUIDE FOR ADOLESCENT**

1. **Interview guide for pregnant adolescent**

**Identification Code: - …………………………………**

1. **Biographical history**

- Kindly tell us about your family background
- *Tueleze kuhusu familia yako*
- What level of education did you attain(Reason)
- *Ulisoma hadi kiwango gani (Sababu ya kukatiza masomo)*

1. **Circumstances leading to pregnancy**

- At what age did you have your first sexual experience
- *Ulishiriki ngono mara ya kwanza ukiwa miaka ngapi*
- Please elaborate on the circumstances that led to that experience
- *Je,ni sababu zipi ziliusika na uamuzi huo*
- Would you tell us about your personal experience with pregnancy
- *Tuelezee hisia zako kuhusu uja uzito wako*
- What was the reaction of your boyfriend/ partner (s) and immediate family when you announced your pregnancy
- *Mume aliyehusika na familia yako ilichukulia vipi uja uzito wako*
- Would you tell us how your neighbours, relatives and friends treated you after learning about your pregnancy
- *Majirani,jamaa na marafiki walichukulia vipi uja uzito wako*

1. **Challenges faced with the pregnancy**

- Economic challenges (Food, clothing, shelter, access to medical services /*Changamoto za lishe,mavazi,makao na huduma za afya)*
- Social challenges(Social support, level of education, domestic violence, sexual abuse and alcohol/substance abuse)/ *Changamoto ya kijamii*
- Medical challenges (STI/HIV,mental illnesses*)/Magonjwa ya zinaa na Changamoto ya kisaikologia*

1. **Antenatal Depression**

- What is your understanding of the term depression
- *Unaelewa vipi ugonjwa wa unyongovu*
- What is your experience with depression
- *Umewahi kuathirika na unyogovu*

1. What can girls in your situation do to cope with depression

*Ni mawaidha gani unaweza kupeana kwa wasichana walioathirika na uyongovu*

6*.* Thank you for your time. Kindly note that a referral note is available for further treatment and follow-up at the Kenyatta National Hospital Youth Centre. The services offered are free of charge and we recommend that you consult them.

*Ahsante sana kwa wakati wako. Matitabu bila malipo yapatikana katika kliniki ya vijana ilioko hosipitali kuu ya Kenyatta. Tunakusihi upate matibabu na ushauri kutoka kwao.*
