# Supplementary material for: Adversities and mental health needs of pregnant adolescents in Kenya: identifying interpersonal, practical, and cultural barriers to care
Source: BMC Womens Health. 2018 Jun 15;18:96. doi: 10.1186/s12905-018-0581-5 (PMC6003032; doi:10.1186/s12905-018-0581-5)
Supplement: Supplementary file 2 — Interview guide with caregivers and partner (DOCX 22 kb) [file 12905_2018_581_MOESM2_ESM.docx]

**ADDITIONAL FILE II Interview guide for the caregiver**

**Identification**

**Code: - …………………………………**

1. **Biographical history**

- Kindly tell us about your family background
- *Tueleze kuhusu familia yako*
- What level of education did you attain(Reason)
- *Ulisoma hadi kiwango gani (Sababu ya kukatiza masomo)*

1. **Possible circumstances that led to your daughter’s pregnancy**

- Please elaborate on the circumstances that may have led to her pregnancy
- *Kwa maoni yako , ni jambo lipi lililosababisha matokeo hayo*
- Would you tell us about your reaction to her pregnancy
- *Tuelezee hisia zako kuhusu uja uzito wa mtoto wako*
- What was the reaction of your partner (s) and immediate family when they discovered she is pregnant
- *Mume wako and familia yako kwa jumla ilichukulia vipi uja uzito wake*
- Would you tell us how your neighbours, relatives and friends treated her after learning about of her pregnancy
- *Majirani,jamaa na marafiki walichukulia vipi uja uzito wako*

1. **Current challenges posed by your daughter’s pregnancy**

- Economic challenges (Food, clothing, shelter, access to medical services /*Changamoto za lishe,mavazi, makao na huduma za afya)*
- Social challenges(Social support, level of education, domestic violence, sexual abuse and alcohol/substance abuse)/ *Changamoto ya kijamii*
- Medical challenges (STI/HIV,mental illnesses*)/Magonjwa ya zinaa na Changamoto ya kisaikologia*

1. **Antenatal Depression**

- What is your understanding of the term depression
- *Unaelewa vipi ugonjwa wa unyongovu*
- What is your experience with depression
- *Umewahi kuathirika na unyogovu*

1. Would you tell us about your experience(personal or with neighbours) with adolescent pregnancy or ante-natal depression

*Mawaitha gani unaweza kupeana kwa wasichana walioathirika na uyongovu*

6*.* Thank you for your time. Kindly note that a referral note is available for further treatment and follow-up at the Kenyatta National Hospital Youth Centre. The services offered are free of charge and we recommend that you take your daughter to this centre.

*Ahsante sana kwa wakati wako. Matitabu bila malipo yapatikana katika kliniki ya vijana ilioko hosipitali kuu ya Kenyatta. Tunakusihi upeleke msichana wako apate matibabu na ushauri kutoka kwao.*

**Ta**

**Sample description (only for review purpose)**

| **Adolescent participants N=12 Mean age = (16.8 ) Mean gestational age =6.08** | | | | | | | | | | | | | | | | | | | | | |
| --- | --- | --- | --- | --- | --- | --- | --- | --- | --- | --- | --- | --- | --- | --- | --- | --- | --- | --- | --- | --- | --- |
| **Participant** | **Pregnancy accepted by parents** | | | **Depression severity on PHQ-9** | **Living alone/**  **family** | **Spouse/**  **boyfriend support** | | | **Food insecurity** | **Paucity of resources for health and antenatal care** | | | **HIV +** | **Domestic or intimate partner violence** | | | **Commeri-cal sex work** | **Schooling continued** | **Financial insecurity** | **Gestational age of baby** | |
| 1 | No | | | 20 | Family | No | | | Yes | Yes | | | No | No | | | No | No | Yes | 7 months | |
| 2 | Yes | | | 19 | Family | No | | | Yes | Yes | | | No | No | | | No | No | Yes | 9 months | |
| 3 | No | | | 15 | Alone | No | | | Yes | Yes | | | Yes (Not onARV- | No | | | Yes | No | Yes | 8 months | |
| 4 | Parents deceased | | | 17 | Partners family | Yes | | | Yes | Yes | | | Yes  on ARV | No | | | No | No | Yes | 4 months | |
| 5 | Married | | | 11 | Married | No | | | No | No | | | No | Yes | | | No | No | Yes | 5 months | |
| 6 | Married | | | 22 | Married | No | | | Yes | Yes | | | No | Yes | | | No | No | Yes | 9 months | |
| 7 | Married | | | 21 | Married | Yes | | | Yes | Yes | | | No | No | | | No | No | Yes | 5 months | |
| 8 | Parents deceased | | | 9 | Alone | No | | | Yes | Yes | | | No | No | | | No | No | Yes | 4 months | |
| 9 | Married | | | 22 | Married | Yes | | | No | Substance(Khat) abuse | | | No | No | | | No | No | No | 4 months | |
| 10 | No | | | 7 | Family | Yes | | | No | No | | | No | No | | | No | Yes | Yes | 5 months | |
| 11 | Married | | | 3 | Married | Yes | | | Yes | Yes | | | No | No | | | No | Yes | Yes | 7 months | |
| 12 | Yes | | | 24 | Family | No | | | No | No | | | No | No | | | No | Yes | No | 6 months | |
|  | **Caregiver sample N=4, 3 mothers (mean age =40), one male partner of the adolescent (age =23 )** | | | | | | | | | | | | | | | | | |  |  | |
|  |  | | | | | | | **Food and income insecurity** | | | | **Social stigma** | | | **Worry about future of adolescents** | | | | **Support from her partner/family** | | |
| Mother of participant 1 | | 31years | Married with 3 other young children | | | | Yes, Unemployed and living with pregnant daughter’s step-father. | | | | Mother angry, was a pregnant teen, Grand-parents previously living with pregnant daughter returned her to her mother. | | | | | Exploring avenues for adoption so that the pregnant daughter can go back to school. | | | No, partner declines to discuss issue | |  |
| Mother of participant 2 | | 40 years | Single mother with 2 other children. | | | | Financial constraints, children dropped out of school, doing manual jobs. Not prepared for the extra expenses | | | | Extended family (grandfather angry)Mother expressed hopelessness at the situation but decided to support daughter | | | | | Daughter dropped out of school, mother intends to help daughter secure manual jobs. | | | None | |  |
| Mother of participant 12 | | 48 years | Married, daughter an only child delivered after 2 miscarriages and childlessness. Church counselor | | | | Parents gainfully employed | | | | Parents and grandparents supportive, but teen has self-stigma(feels let down family) | | | | | Going back to school after delivery, parents willing to raise the child. | | | Father expressed disappointment initially but now co-operative | |  |
| Partner of participant 4 | | 23 years | Un-employed living with the mother and his siblings | | | | Dependent on the mother. Expresses frustration when not able to care for his wife and the unborn child, | | | | Inability to care for his immediate family. | | | | | Couple tested HIV positive. On counseling and ARV treatment. Declined audio-recording. | | | Supports and accompanies wife to clinic | |  |
